# Identification of molecular processes needed for vascular formation through transcriptome analysis of different vascular systems

**DOI:** 10.1186/1471-2164-14-217

**Published:** 2013-04-02

**Authors:** Peng Xu, Yimeng Kong, Xuan Li, Laigeng Li

**Affiliations:** 1 National Key Laboratory of Plant Molecular Genetics and Key Laboratory of Synthetic Biology, Institute of Plant Physiology and Ecology, Chinese Academy of Sciences, 300 Fenglin Rd, Shanghai 200032, China

## Abstract

**Background:**

Vascular system formation has been studied through molecular and genetic approaches in *Arabidopsis*, a herbaceous dicot that is used as a model system. Different vascular systems have developed in other plants such as crops and trees. Uncovering shared mechanisms underlying vascular development by transcriptome analysis of different vascular systems may help to transfer knowledge acquired from *Arabidopsis* to other economically important species.

**Results:**

Conserved vascular genes and biological processes fundamental to vascular development were explored across various plants. Through comparative transcriptome analysis, 226 genes from *Arabidopsis*, 217 genes from poplar and 281 genes from rice were identified as constituting 107 conserved vascular gene groups. These gene groups are expressed mainly in vascular tissues and form a complex coexpression network with multiple functional connections. To date, only half of the groups have been experimentally investigated. The conserved vascular gene groups were classified into 9 essential processes for vascular development. 18 groups (17%) lack of annotations were classified as having unknown functions.

**Conclusion:**

The study provides a map of fundamental biological processes conserved across different vascular systems. It identifies gaps in the experimental investigation of pathways active in vascular formation, which if explored, could lead to a more complete understanding of vascular development.

## Background

The plant vascular system, which includes xylem and phloem tissues, connects different parts of the plant body and is of great importance for mechanical support as well as the transport of water, nutrients and photosynthesized compound. Monocots and dicots have developed different vascular systems [1]. Most monocots, which produce scattered vascular bundles, only develop primary growth, whereas woody plants usually develop secondary vascular systems through secondary growth. Several stages of development are common across different vascular systems including: differentiation of procambium or cambium cells, elongation of tracheary elements and sieve cells, and secondary wall formation in vascular sclerenchyma cells [2]. These shared processes suggest vascular development may be regulated by a set of underlying molecular mechanisms.

*Arabidopsis thaliana*, a typical herbaceous dicot, has been employed as a model plant to study the molecular mechanisms regulating the initiation, development and regulation of the vascular system. A number of important transcriptional regulators have been discovered using the *Arabidopsis* model system [3]. For example, *MONOPTEROS* (*MP*) is an auxin response factor required for the establishment of vascular tissues [4]. *APL* encodes a MYB transcription factor crucial for phloem identity in the root [5]. *HD-ZIPIII*s and *KAN*s are two distinct classes of transcription factors that regulate differentiation of xylem and phloem tissues from procambium cells [6]. *VND6* and *VND7*, two NAC family genes, promote xylem vessel cell formation [7]. *SND1* and *NST1*, also members of the NAC family, regulate secondary wall biosynthesis [8]. The identification of these molecular mechanisms in *Arabidopsis* may be used to shed light on analogous processes in monocots and woody plants.

Recent research suggests genes involved in the same pathway tend to be transcriptionally coordinated [9-11]. Transcriptome profiling and analysis of gene coexpression can uncover functional connections as well as identify molecular pathways between genes [12-14]. For example, networks involved in cell wall formation were identified through coexpression analysis of transcriptomes across different plant species [15,16]. Here we report an analysis of the transcriptomes in three types of vascular plants (*Arabidopsis*, poplar and rice) to identify the essential genes and processes needed for vascular development.

## Results and discussion

### Identification of conserved vascular genes across various vascular systems

**Figure 1:**
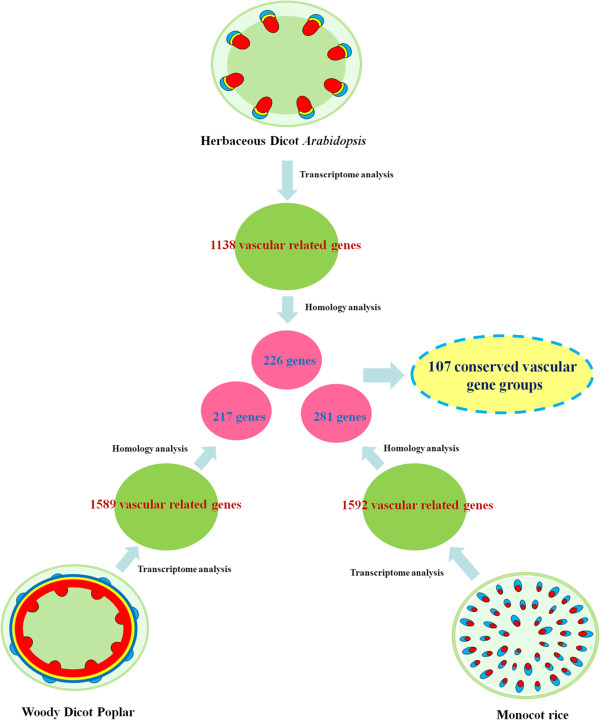
**Identification of conserved vascular gene groups across different vascular systems.** Microarray data from *Arabidopsis*, poplar and rice were analyzed for classification of vascular related genes. By homology analysis, conserved vascular genes in three species were identified, which form 107 groups. Homology analysis between species is performed by BLAST program with E ≤ 1e-50.

Vascular associated genes from three vascular systems were identified by comparative transcriptome analysis. Microarray data from 8 *Arabidopsis* tissues, 9 poplar tissues and 8 rice tissues were analyzed (see Methods and Additional file 1). In each species, vascular associated genes satisfied two criteria. First, its expression value in the stem had to be higher than in any other tissue (P-value < 0.05) and second, its expression value had to be at least twice as high as the geometric mean value of all tissues analysed. Consequently, 1138 genes (5.0% of the total pool of genes with a unique probe) in *Arabidopsis*, 1589 genes (5.8%) in poplar and 1592 genes (6.2%) in rice met the criteria (Figure 1).

The identified vascular associated genes were further analyzed in parallel for the presence of orthologs across the three vascular systems. 226 genes in *Arabidopsis*, 217 genes in poplar and 281 genes in rice were identified to be conserved across the three species by the BLAST program with E-value cut-off of 1e-50 (Figure 1 and Additional file 2). These conserved vascular genes (CVGs) can be categorized into 107 groups (Figure 1), consisting of sets of orthologs performing functions which may be essential to vascular development (Additional file 3).

### CVGs are preferentially expressed in vascular tissues

**Figure 2:**
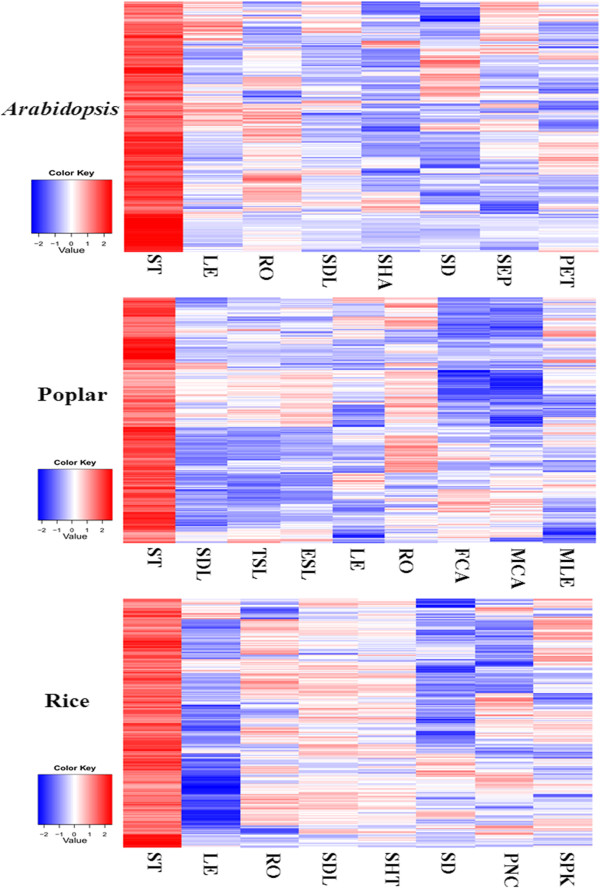
**Expression profiles of CVGs in various tissues. **Microarray data of CVGs in different tissues were calculated and standardized. Heatmaps are generated for *Arabidopsis*, poplar and rice separately. ST - stem; LE - leaf; RO - root; SDL - seedling; SHA - shoot apex; SD - seed; SEP - sepals; PET - petals; TSL - transferred seedling; ESL - etiolated seedling; FCA - female catkin; MCA - male catkin; MLE - mature leaf; PNC - panicle; SPK - spikelet.

Heatmaps were generated from the CVG expression values (Additional file 2) after normalization across various tissues (Figure 2). CVG expression was enriched in the stem, moderate in the root, and low in leaves, a pattern reflecting their primary activity in the vascular system. Since vascular tissues contain different cell types, we further tested whether CVG expression is enriched in xylem, phloem or cambium cells. 64 of the 226 CVGs detected in *Arabidopsis* were examined in a previous study (Additional file 4) [17]. 63 of those CVGs were found to be expressed in xylem, phloem or cambium. 49 of the 63 genes showed preferential expression within vascular tissues toward xylem versus 5 genes toward phloem-cambium, indicating that xylem development may involve more CVGs than phloem.

### CVGs form a complex coexpression network

**Figure 3:**
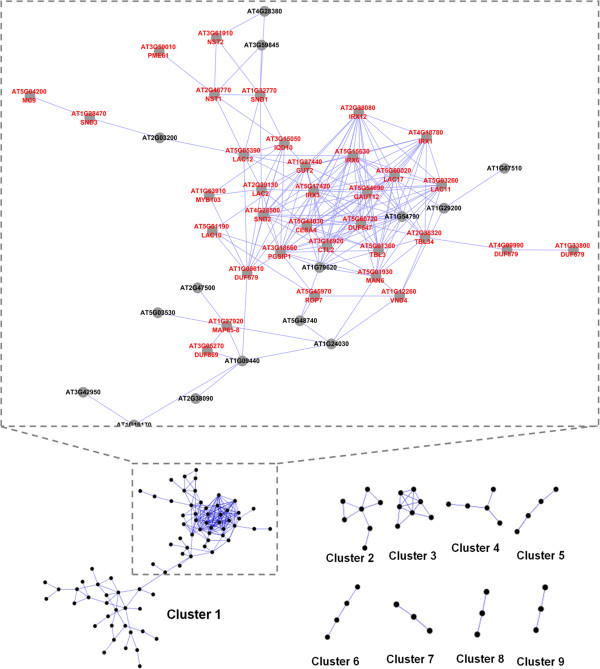
**Coexpression network formed by CVGs. **115 CVGs form a coexpression network which contains 9 clusters. Enlarged picture shows a part of cluster 1 for detailed illustration. Nodes with gene annotations are indicated by red color.

The vascular CVGs were analyzed through coexpression analysis programs [14,18]. 115 of the 226 CVGs in *Arabidopsis* displayed coexpression relationships in a complex network (Figure 3). These CVGs formed 9 clusters, indicating that a coordinated system of multiple networks is involved in vascular development. Of the 9 clusters, cluster 1 is the largest and consists of 79 genes (Additional file 5). Both cluster 2 and cluster 3 consist of 7 genes. Cluster 4 has 5 genes and the remaining clusters contain 3 or 4 genes.

Although many transcription factors have been identified as regulators of vascular development, their signal transduction networks remain largely uncharacterized [3]. Specific genes stood out in cluster 1 due to their association with key transcription factors. Transcription factors *SND1*, *SND2*, *NST1* and *MYB103* are connected in cluster 1 (Figure 3). They are closely coexpressed with genes involved in secondary wall biosynthesis, consistent with the central role that these transcription factors play in regulating secondary wall formation [8,19,20]. Interestingly, *IQD10* (*AT3G15050*) is also connected in the cluster forming direct links with *SND1*, *NST1* and *MYB103*. The function of *IQD10* is unknown. Electronic annotations suggest *IQD10* contains a plant-specific IQD domain and is a putative calmodulin target protein mediating calcium signals [21]. The coexpression analysis suggests *IQD10* is likely involved in signaling pathways needed for secondary cell wall formation.

*AT5G60720*, which contains a domain of unknown function (DUF) is another intriguing gene in cluster 1. *AT5G60720* is closely coexpressed with genes involved in secondary wall biosynthesis such as *IRX1*, *IRX3*, *IRX6*, *GUT2*, *GAUT12* and *LAC2 *[22-25]. Secondary cell walls contain mainly cellulose, hemicellulose and lignin. The biosynthesis and modification of these carbohydrate-based cell wall components involves highly coordinated processes among a large suite of genes with specialized functions [26]. *AT5G60720* may play a novel, yet to be elucidated role in the process of secondary cell wall biosynthesis.

Cluster 1 also includes *ROP7*, which belongs to a family of Rho-related GTPase (ROP) that is critical for regulating cytoskeleton organization in plants [27]. ROP family genes have been speculated to participate in vascular development by regulating secondary wall patterns formation in xylem [28]. GFP-ROP7 was found to be localized at the plasma membrane in differentiating xylem cells in culture. Histochemical analysis of ROP7: GUS demonstrated that *ROP7* is preferentially expressed in developing xylem cells with partially thickened secondary walls [29]. Here, *ROP7* is found in the coexpression cluster with secondary wall biosynthesis genes, supporting its potential functions during secondary wall formation. Cluster 1 also contains several protein kinases and leucine-rich repeat protein kinases (Additional file 5) which are typically involved in signal perception and mediation [30]. Their appearance suggests the presence of signal transduction pathways in vascular development which have yet to be investigated.

**Figure 4:**
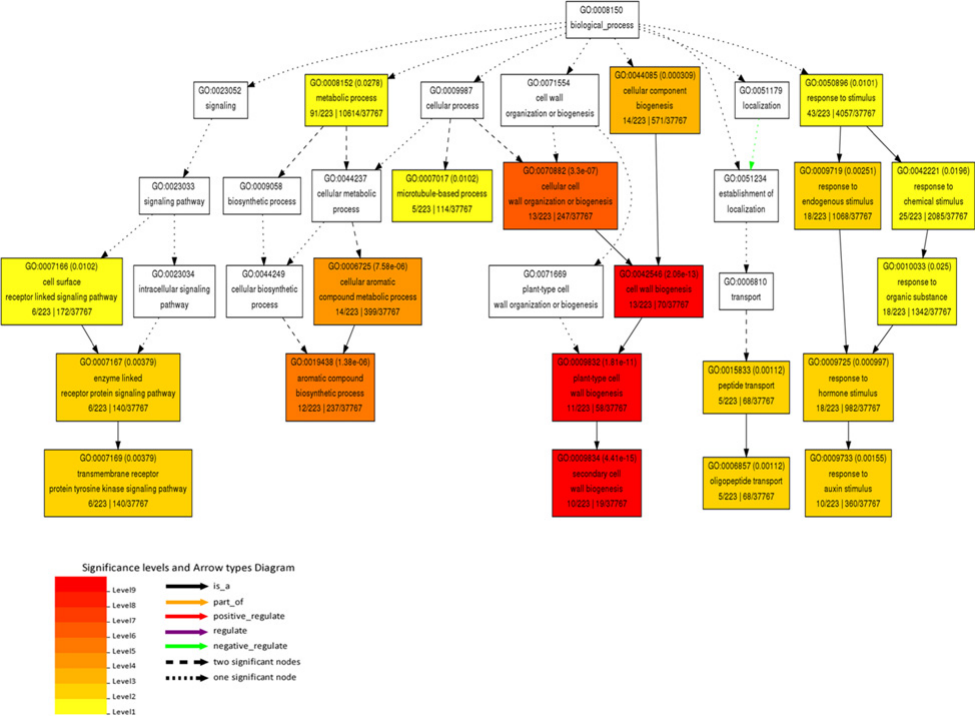
**Biological processes enriched in the CVGs. **CVGs in *Arabidopsis *are analyzed by agriGO for GO enrichment. Enriched GO terms with Fisher test FDR less than 0.05 are considered as significant and indicated by corresponding color levels. GO terms, FDR and GO descriptions are shown in each significant node.

The 7 members of cluster 3 include *4CL*, *CCR1*, *CAD5*, *C3H*, *OMT1*, *PAL1* and *PAL3* (Additional file 4). These genes encode key enzymes which catalyse the biosynthesis of monolignols [31]. This suggests lignin biosynthesis is conserved across the different vascular systems. However, since the monolignol catalysing genes from cluster 3 are less coexpressed with cellulose and xylan biosynthesis genes in cluster 1 (Figure 4), the two biosynthesis pathways are likely have different regulatory mechanisms.

### Enriched GO terms of CVGs reflect important cellular and biochemical processes

Vascular development includes several critical stages including polarized cell differentiation, cell elongation and secondary wall formation [2]. GO analysis indicates that CVG activity is significantly enriched (FDR cut-off at 0.05) in the following processes: cellular component biogenesis, metabolic process, microtubule-based cell organization, peptide transportation, and cell surface receptor linked signaling processes (Figure 4).

Cellular component biogenesis includes secondary cell wall biosynthesis, an outstanding feature during vascular development. Metabolic process is mainly related to aromatic compound biosynthesis responsible for lignin formation. These two enriched processes indicate the biosynthesis of lignin and secondary wall components are both highly conserved in vascular development. Peptide transport and microtubule-based processes are responsible for protein transport and cytoskeleton organization, which are crucial for cell wall formation and cell development. Cell surface receptor linked signaling pathway is another enriched process, indicating busy signal transduction during vascular formation. Mapping CVGs to their enriched GO terms provides a basic roadmap of the important processes involved in vascular development.

### CVG groups represent 9 fundamental processes during vascular development

**Figure 5:**
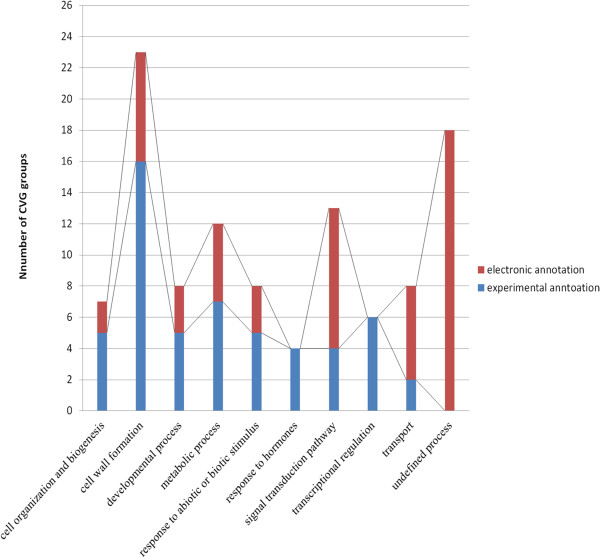
**The fundamental processes conducted by CVG groups. **107 CVG groups participate in 9 fundamental processes according to their experimental annotations or electronic annotations. CVG groups without sufficient description are classified into the undefined process.

Experimental descriptions were used to link CVG groups to general biological processes. Of the 107 CVG groups, only a half (54 groups) has experimental annotations (GO evidence code IDA, IEP, IPI, IMP, and IGI) which can be categorized into 9 fundamental processes (Figure 5): cell organization and biogenesis, cell wall formation, developmental process, metabolic process, response to abiotic and biotic stimulus, response to hormones, signal transduction pathway, transcriptional regulation and transport. The other 53 groups have not been described experimentally and only have electronic annotations (Additional file 3). Among these, 35 fall into the 9 existing processes while 18 groups (17% of total CVG groups) are involved in undefined processes (Figure 5).

### Transcriptional regulation

**Figure 6:**
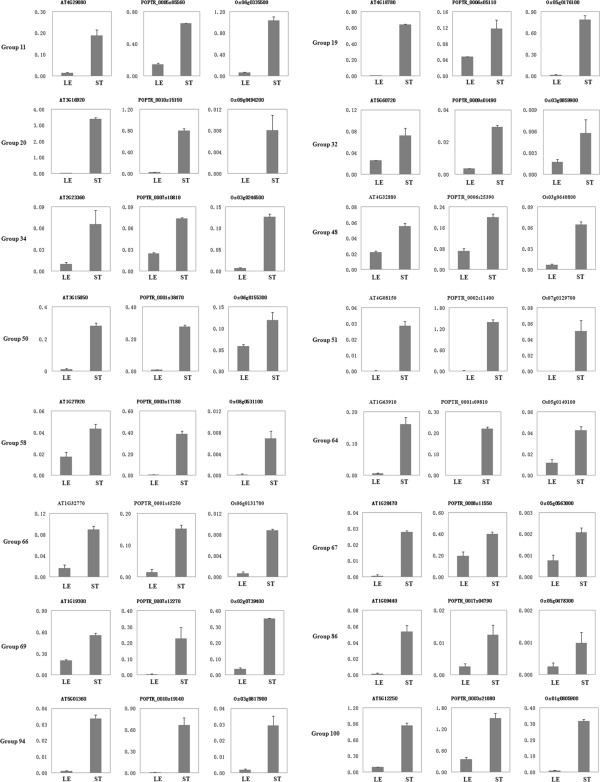
**Expression determination of 16 CVG groups in various tissues. **Total RNAs were isolated from the stems and leaves of *Arabidopsis*, poplar and rice for qRT-PCR analysis. Expressions of 16 CVG groups, including 48 genes are analyzed. Triplicate repeats were performed and relative expression values were normalized with respect to ubiquitin reference gene.

6 CVG groups which have experimental annotations fall in the category of transcriptional regulation process. Among them 5 CVG groups encode transcription factors: *HD-ZIP III*s, *KNOX*s, *NAC*s and *MYB*s, respectively (Additional file 3). Their vascular-enriched expression pa-ttern was verified by qRT-PCR analysis (Figure 6, Group 48, 51, 64, 66 and 67). In *Arabidopsis*, *HD-ZIP III*s display a complex pattern of overlapping functions [32] and regulate vascular patterning by promoting the differentiation of procambium cells into xylem [33,34]. In poplar, *HD-ZIPIII* genes *POPCORONA* and *popREVOLUTA* play a role in regulating cell differentiation and patterning of vascular tissues [35,36], while *PtrHB7* regulates secondary vascular differentiation in a dosage dependent manner [37]. *BREVIPEDICELLUS* (*BP*), which is a member of the *KNOX* gene family in *Arabidopsis*, maintains cells in an indeterminate state through negatively regulating secondary cell wall metabolism [38]. Similar functions were observed in a poplar ortholog, *ARBORKNOX2* (*ARK2*). Overexpression of *ARK2* showed delayed cell differentiation and misregulation of cell wall biosynthesis-related genes. Suppression of *ARK2* enhanced development of secondary xylem and phloem fibers [39]. These indicate *KNOX* family members are conserved and necessary for cell differentiation. *NAC* and *MYB* are transcription factors that play a key role in activating the secondary wall biosynthetic program in *Arabidopsis*[8,19]. NACs and MYBs have also been shown to regulate secondary wall biosynthesis in trees such as poplar and *Eucalyptus*, and in grass species including rice and maize [40,41]. As expected, our results also indicate that NACs and MYBs are crucial transcriptional regulators in the three vascular systems and perhaps throughout vascular plants.

### Signal transduction pathway

Only 4 of the 13 CVG groups involved in the signal transduction pathway have been experimentally described. The CVGs include several signal pathway components including PXY, a receptor of polypeptide signaling molecule CLE41 [42]. In *Arabidopsis*, PXY functions in meristematic cells of procambium. Its knockout mutant displays loss of ordered cell division patterns in procambium zone [43]. The binding of polypeptide CLE41, which is secreted from phloem, to PXY is believed to regulate the orientation of procambium cell division [44]. The interaction between PXY and CLE41 is likely to be a common mechanism to promote polarized cell division. In addition, the CVGs include *IQD10* which is involved in calcium signaling. Coexpression analysis indicates it networks with secondary wall biosynthetic genes. A homologous gene, *IQD1*, was found to be calmodulin-binding and mainly expressed in vascular tissues [45]. Quantitative RT-PCR analysis of *IQD10* confirmed its enriched expression pattern in vascular tissues (Figure 6, Group 50). These results suggest calcium signaling may be involved in vascular development. Continued characterization of the CVGs will help to identify additional signal transduction pathways embedded in vascular development.

### Cell wall formation

Cell wall formation includes 23 CVG groups (16 have experimental annotations), reflecting the fact that cell wall biosynthesis is a major process during vascular development. *IRX1*, *IRX3* and *IRX5* in Group 19 encode cellulose synthase (CesA) which catalyses cellulose synthesis in cell walls [22]. Hemicellulose biosynthesis genes, *PARVUS*, *GUT2* and *GAUT12*[23,24,46,47], and lignin biosynthesis genes, *4CL*s and laccases [25,31] are also contained in the cell wall formation process. *GUT2*, *GAUT12* and laccases form a closely coexpressed cluster, highlighting their critical roles in cell wall biosynthesis. In addition, several CVGs such as chitinase-like proteins and pectinesterases are particularly involved in primary wall formation. Quantitative RT-PCR analysis reinforces the conserved roles of the CVGs in diverse vascular systems (Figure 6, Group 19 and 20).

### Response to hormones

4 CVG groups involved in response to hormones have experimental annotations (Additional file 3). Group 11 encodes short-lived transcriptional repressor Aux/IAA that interacts with auxin response factors [48,49]. According to the auxin canalization hypothesis, vascular initiation is regulated by directional auxin flows [50]. Hence, Aux/IAAs are likely to be involved in the response of early auxin signals during vascular system initiation. Response to auxin stimulus is enriched in above GO analysis and several auxin response factors are included in CVGs (Additional file 3). This indicates the likelihood that a conserved mechanism exists to mediate the detection of auxin signals. The proposed role of *Aux/IAA* orthologs in the vascular development of the three species is supported by quantitative analysis of their expression patterns (Figure 6, Group 11).

### Cell organization and biogenesis

Quantitative expression analysis demonstrated Group 58 genes are transcriptionally enriched in vascular systems (Figure 6). Microtubule-associated protein 65 (*MAP65*) from Group 58 is associated with cell organization and biogenesis. Critical functions of *MAP65* can be deduced from *ZeMAP65-1*, a homologous gene preferentially expressed in xylogenic Zinnia mesophyll cells. Overexpression of *ZeMAP65-1* in *Arabidopsis* suspension cells induces dramatic changes in cortical microtubule bundles [51]. This suggests MAP65 family genes may play a conserved role in assembly of microtubules during tracheary elements formation.

### Other fundamental processes

The fundamental processes concluded from GO analysis also include developmental process (8 CVG groups), metabolic process (12 CVG groups), transport (8 CVG groups), and response to abiotic or biotic stimulus (8 CVG groups). Although enriched and conserved across the three vascular systems, these CVG groups may not directly participate in vascular development. For example, CVGs in metabolic process include enzymes from various biochemical pathways (Additional file 3), which may indirectly affect the vascular development.

### Undefined process

18 CVG groups had uncharacterized functions and were classified under the undefined process. Although the roles of these genes have yet to be determined, the potential for them to play an important role in vascular development should not be overlooked. For example, the groups include several DUF-containing proteins (Additional file 3) such as *DUF547* and *DUF869* which are preferentially expressed in vascular tissues (Figure 6, Group 32 and Group 34). Recently, the DUF579 gene family was reported to play a role in xylan biosynthesis during cell wall formation. Mutants of two DUF579 genes exhibited distorted xylem vessels as a result of reduced xylan content [52]. A member of the DUF579 family was shown to encode a glucuronoxylan methyltransferase that catalyzes 4-O-methylation of the glucuronic acid substituents [53]. Like DUF579 proteins, the CVG groups in the undefined process may play essential roles in the 9 fundamental processes of vascular development.

## Conclusions

We used comparative transcriptome analysis to identify the essential genes and biological processes underpinning vascular development. 107 genes, which can be categorized into 9 fundamental processes, were found to be conserved across three different vascular systems. These results can help to facilitate the transfer of learnings from *Arabidopsis* to different grass and tree species. In addition, our analysis surfaced many conserved genes whose functions have yet to be characterized experimentally. A more comprehensive mapping of the principal mechanisms underlying vascular formation could reveal new avenues for the engineering of plants with desired body structure and improved biomass production.

## Methods

### Microarray analysis

Microarrays from different tissues of *Arabidopsis*, poplar and rice are listed in Additional file 1. Developmental series data of *Arabidopsis* were acquired from NASCArrays. Microarray data of poplar and rice were obtained from NCBI Gene Expression Omnibus (GEO) database under accession numbers GSE13990, GSE21480 and GSE19024. In each species, microarray data were normalized across all arrays using Robust Multiarray Averaging (RMA) method in Bioconductor. Differentially expressed genes were identified by Linear Models for Microarray Data (LIMMA). Expression level of vascular related genes in the stem was confined to those higher than any other tissue with an adjusted P-value less than 0.05 and at the same time at least twice as high as the geometric mean of all tissues. In each species, probe sets of microarrays were converted into gene loci with reference to support documents from Affymetrix website (June 2012). The lasted versions (June 2012) of protein sequences of *Arabidopsis thaliana* (genome release 9), *Populus trichocarpa* (version 2.2) and *Oryza sativa* (IRGSP/RAP build 5) were obtained from The *Arabidopsis* Information Resource (TAIR), Phytozome, and The Rice Annotation Project (RAP) respectively. BLAST program (version 2.2.26) was used for homology analysis. Vascular related genes are regarded as CVGs only when they have orthologs in the other two species with E-value cut-off of 1e-50. Gene expression values in different tissues were standardized according to previously described methods [54] and heatmap.2 in R program was used for generating heatmaps of CVGs.

### GO enrichment analysis

GO enrichment analysis of CVGs in *Arabidopsis* was implemented by agriGO [55] using complete GO ontology and suggested backgrounds. GO terms with Fisher test FDR less than 0.05 were considered as significant enrichment. Representative GO terms were displayed in each hierarchical process.

### Coexpression network construction

Coexpression relationships of each CVG in *Arabidopsis* were obtained from PlaNet with default parameters [14]. Coexpression network was constructed by Cytoscape [18] (version 2.8.3) with force-directed layout. Clusters with less than 3 nodes were removed from the network.

### Fundamental processes analysis

GOSLIM annotations from TAIR (October 2012) were used for fundamental process analysis. In biological processes, GO evidence code IDA, IEP, IPI, IMP, and IGI were considered as experimental annotations. The other evidence codes were regarded as electronic annotations. A publication (from TAIR) of a representative gene in each CVG group is used as group reference in Additional file 3. CVG groups were classified manually into fundamental processes according to group reference and annotations. Groups with little annotated information are classified into the undefined process.

### RNA isolation and qRT-PCR

Stem and rossette leaves of 7 weeks *Arabidopsis thaliana*, the 6th internode stem and mature leaves of poplar (*P. deltoides* × *trichocarpa*), stem and mature leaves of rice (*Oryza sativa var. japonica*) after flowering, were used for RNA extraction. Total RNA was extracted using EASYspin plant RNA extraction kit (Yuanpinghao) according to manufacturer’s instructions. RNA quality was verified by gel electrophoresis and absorption at 260 nm/280 nm. cDNA synthesis of 0.5 ug RNA was carried out using PrimeScript RT reagent kit (TaKaRa) with oligo dT primer and random 6 mers, following the manufacturer’s protocols. Quantitative RT-PCR primers were designed by QuantPrime [56] with manual inspections (Additional file 6). Triplicate qRT-PCR assays per sample were performed using iQ SYBR Green Supermix (Bio-Rad). The reactions were performed as the following conditions: initial denaturation at 95°C for 1 min, three-step thermal cycling with denaturation at 95°C for 15 s, annealing at 60°C for 15 s and extension at 72°C for 20s for 40 cycles. Melting curve analysis was performed with temperature increasing steps of 0.08°C per second, ranging from 72°C to 95°C. Relative expression of each gene was calculated using ⊿ ⊿ Ct method [57]. A ubiquitin gene (*At3g62250*, *POPTR_0001s44440* and *Os02g0161900*) was used as the reference in three species.

## Competing interests

The authors declare that they have no competing interests.

## Authors’ contributions

PX analyzed the data, conducted experiments and prepared the manuscript. LL directed the research project and composed the manuscript. YK wrote scripts and analyzed the data. XL directed data analysis and edited the manuscript. All the authors have approved the final manuscript.

## Supplementary Material

Additional file 1 **Tissues used for microarray analysis in *Arabidopsis*, poplar and rice.** Click here for file

Additional file 2 Expression values of CVGs in different tissues. Click here for file

Additional file 3 List of CVG Groups. Click here for file

Additional file 4 Enriched expression patterns of CVGs in xylem, phloem or cambium. Click here for file

Additional file 5 Genes used for coexpression network construction. Click here for file

Additional file 6 Primers used for qRT-PCR analysis. Click here for file
