# Supplementary figures and images for: Identification of molecular processes needed for vascular formation through transcriptome analysis of different vascular systems
Source: BMC Genomics. 2013 Apr 2;14:217. doi: 10.1186/1471-2164-14-217 (PMC3620544; doi:10.1186/1471-2164-14-217)

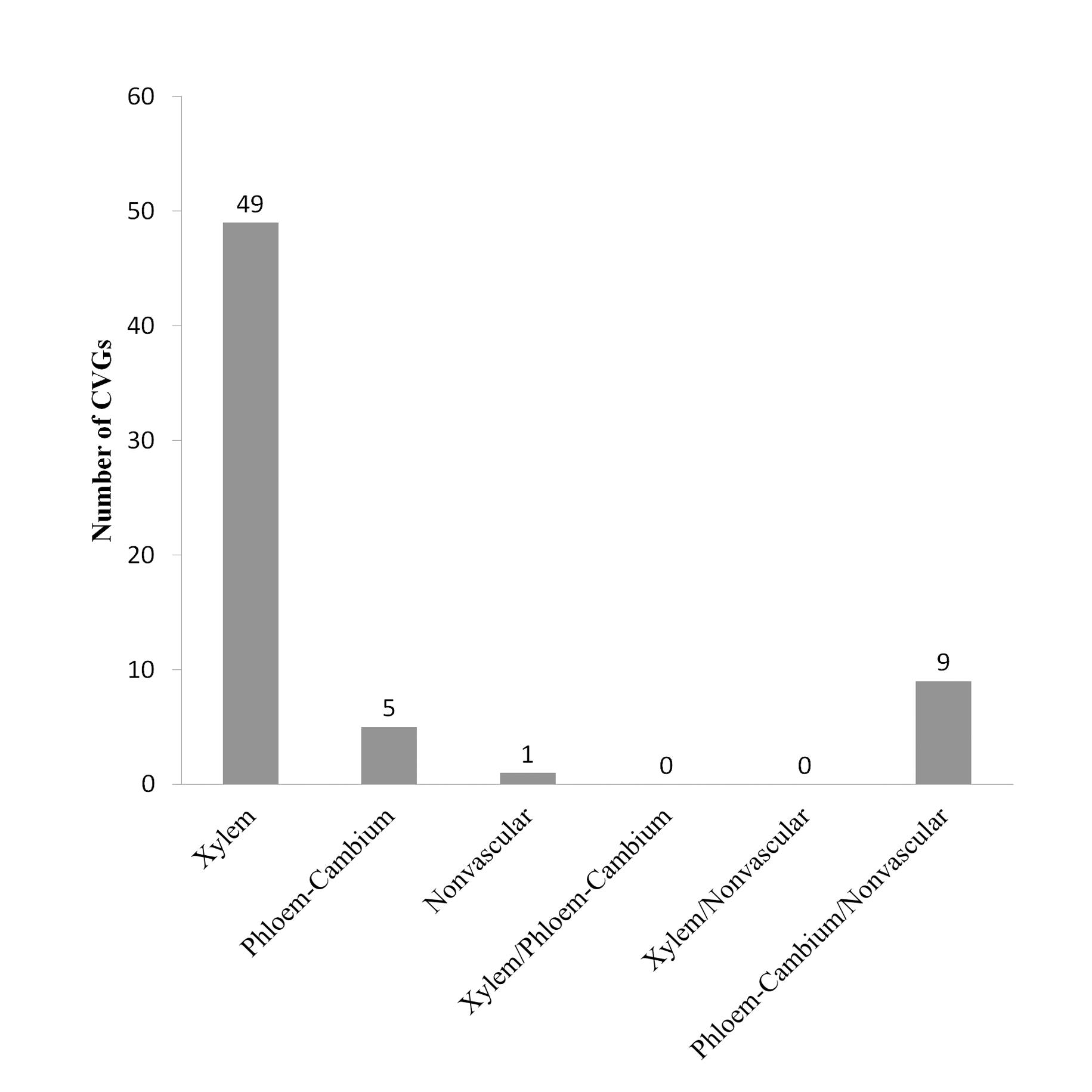

Supplement: Additional file 4 — Enriched expression patterns of CVGs in xylem, phloem or cambium. [file 1471-2164-14-217-S4.tiff]
